# Answer to “Key points of reducing neurologic complications in frozen elephant trunk technique”

**DOI:** 10.5935/1678-9741.20150057

**Published:** 2015

**Authors:** Ricardo Ribeiro Dias, Jose Augusto Duncan

**Affiliations:** Incor, USP – São Paulo, SP, Brazil

Dear Editor,

We appreciated the comments of the colleagues regarding our role and we completely agree with the considerations related to the need of reducing morbidity.

With this in mind, we can say that all points must be addressed in order to prevent morbidity and mortality in our patients.

Recently, we started to operate on this type of surgery in a hybrid operating room, but in the beginning of the learning curve, we did not. Many years ago, when we first started placing stents in the descending aorta, in acute type B dissections, with a Brazilian short endovascular stent graft (9 cm length), which was very smooth and easy to handle, we learned how to do it without a guidewire, which is actually impossible with the current stent grafts. Nowadays, we use the guidewire in almost all cases.

In the beginning of the "Evita Open" experience (the only stent graft available here in Brazil for the frozen elephant trunk procedure), the device had a soft but big "ball" at the end of the prosthesis so as not to harm the dissected layer of the aorta; and for chronic dissections with small true lumen, the pull back traction was also an issue and sometimes it would take several minutes to release the prosthesis. They have changed and now we no longer face this problem.

The stent graft length is not an issue, in my opinion. None was longer than 15 centimeters. In addition, when doing total endovascular procedures, we have covered the entire descending aorta in many cases and we did not have paraplegia.

Cerebral fluid drainage as a spinal cord protection strategy is regularly used, but not for these operations. If you do not have a proximal hypertension hemodynamic situation, which happens in these controlled proximal brain perfusions, there is no reason for cerebral fluid drainage (the situation is completely different from the thoracoabdominal aorta operations).

In conclusion, we can say that with a better and faster surgical procedure, we can regularly do this operation in less than 60 minutes of body circulatory arrest time and no longer have these devastating complications.

Yours sincerely,

**Ricardo Ribeiro Dias, Jose Augusto Duncan** **Incor, USP - São Paulo, SP, Brazil**

